# Age-dependent changes in synaptic plasticity enhance tau oligomerization in the mouse hippocampus

**DOI:** 10.1186/s40478-017-0469-x

**Published:** 2017-09-06

**Authors:** Tetsuya Kimura, Mamiko Suzuki, Takumi Akagi

**Affiliations:** 1 Department of Aging Neurobiology, Center for Development for Advanced Medicine for Dementia, National Center for Geriatrics and Gerontology, 7-430 Omori-cho, Obu-shi, Aichi, 474-8511 Japan; 2 Support Unit for Animal Resources Development, Research Resources Center, Brain Science Institute, RIKEN, Wako, Saitama, 351-0198 Japan; 3 Present Address: Department of Alzheimer’s Disease Research, Center for Development for Advanced Medicine for Dementia, National Center for Geriatrics and Gerontology, 7-430 Omori-cho, Obu-shi, Aichi, 474-8511 Japan

## Abstract

The aggregation mechanism of phosphorylated tau is an important therapeutic target for tauopathies, including Alzheimer’s disease, although the mechanism by which aggregation occurs is still unknown. Because the phosphorylation process of tau is involved in the trafficking of AMPA receptors, which accompanies the long-term depression (LTD) of synapses, we examined the effect of LTD-inducing low-frequency stimulation (LFS) on the formation of pathological tau aggregates in adult and aged wild-type mice. Our biochemical analysis demonstrated that LFS led to the formation of sarkosyl-insoluble (SI) tau oligomers in aged hippocampi but not in adult hippocampi in wild-type mice. In parallel, electrophysiological experiments showed an increased contribution of the autophagy-lysosomal pathway (ALP) to LTD during aging, although the other properties of LFS-induced LTD that we investigated were not altered. Thus, we anticipate that the increased contribution of the ALP to the LTD cascade is involved in the age-dependent formation of tau oligomers that results from LFS. Analysis of the LC3 ratio, an indicator of autophagosome formation, showed that LFS increased cleaved LC3 (type II) in the aged hippocampus relative to type I LC3, suggesting potentiation of the ALP accompanied by LTD. Pharmacological inhibition of autophagosome formation depressed LFS-induced oligomerization of tau. Prevention of lysosomal function in the ALP enhanced the formation of tau oligomers by LFS. These results suggest the importance of the autophagosome for the LFS-induced oligomerization of tau and suggest a reason for its age dependency. Interestingly, the lysosomal disturbance promoted the formation of the fibrillar form of aggregates consisting of hyper-phosphorylated tau. The LTD-ALP cascade potentially acts as one of the suppliers of pathological aggregates of tau in aged neurons.

**Electronic supplementary material:**

The online version of this article (10.1186/s40478-017-0469-x) contains supplementary material, which is available to authorized users.

## Introduction

The brains of individuals with Alzheimer’s Disease (AD) are characterized by two anatomical hallmarks, beta-amyloid (Aβ)-containing senile plaques and neurofibrillary tangles (NFTs), which consist of twisted fibers of the protein tau. Although most people spontaneously develop NFTs as they age, those with AD tend to develop far more [4]. Importantly, the degree of dementia in AD patients is highly correlated with the frequency of NFTs [30]. Thus, understanding the mechanisms of the sporadic development of tau aggregates is necessary to produce effective therapeutic strategies for dementia in sporadic AD. To date, these mechanisms have been poorly understood.

Some studies have examined, both in vitro and in vivo, the possible role of tau phosphorylation on its aggregation [31, 33, 43], suggesting that under some conditions tau phosphorylation may increase its capacity for self-assembly [2]. The cellular ability to carry out protein degradation also affects tau accumulation and aggregation. For example, proteasomal inhibition increases the total level of tau and facilitates the formation of detergent-protective tau aggregates in rat brains [25]. In addition, tau phosphorylation is a signal for its degradation by the ubiquitin-proteasome system [23]. Perturbation of autophagy also enhances tau aggregation in a cell model consisting of overexpressed human tau [11]. Furthermore, the stimulation of autophagy ameliorates tau pathology in tau-overexpressing mice, also indicating the involvement of autophagy in tau degradation [39]. NFTs are found in Niemann-Pick disease type C, which affects lysosomal function, suggesting that impairment of lysosomes might be one of the causes of tau aggregation [52].

Tau is categorized as a microtubule-associated protein and contributes to the stabilization of the cytoskeleton [12] and to neuronal development [5, 44]. Classically, tau has been considered to be involved in axonal functions, such as axonal transport of molecular cargo [9], because of its stable localization in axons [3]. Recent studies, however, have demonstrated the activity- or transmitter- dependent expression of tau in the somato-dendritic regions of neurons [22, 49]. Therefore, tau was also expected to be involved in dendritic functions, as was shown by several groups that reported a critical role for tau in a form of synaptic plasticity, long-term depression (LTD) [20, 27, 37]. These studies showed that phosphorylation of tau is promoted by LTD-inducing stimuli [20, 27] and that phosphorylated tau is required for the formation of LTD [37]. Additionally, a study using the overexpression of wild-type human tau in mice showed that age-dependent accumulation of phosphorylated tau in the somato-dendritic region was negatively correlated with spine number and neural activity [19]. Furthermore, in AD brains, a similar negative correlation between the accumulation of phosphorylated tau and spine density has been reported [26]. Those studies suggest that the phosphorylation state of tau influences dendritic functions, such as synaptic functions, in adult and aged brains.

In the present study, we examined whether the tau modification processes that accompanies LTD contributes to the formation of detergent-protective tau aggregates, sarkosyl-insoluble (SI) tau [24]. We found that LFS formed SI tau aggregates in an age-dependent manner in vivo. Furthermore, the mechanism that leads to the age dependency of tau oligomerization was examined using electrophysiological, biochemical, and pharmacological techniques.

## Materials & methods

### Animals

C57/BL6J mice were used for all experiments except where otherwise noted. Tau-deficient mice (Mapt^tm1Hnd^/J, The Jackson Laboratory) were maintained by backcrossing with C57/BL6J mice. All mice were kept on a 12-h light/12-h dark cycle at 23 °C and had free access to food and water. In the present study, only male animals were used. Mice were divided into two age categories: adult mice, which were 4–10 months old and aged mice, which were 20–24 months old. More detailed age ranges of the animals used in each experiment are described in the Results.

### Drugs and antibodies

The drugs used in this study were as follows: picrotoxin (Sigma), a GABA-receptor inhibitor; two different autophagy inhibitors, 3MA (3-methyladenine; Santa Cruz Biotechnology) and Bafilomycin (Bafilomycin A1; AdipoGen); and the proteasome inhibitor MG132 (Chemscene). The antibodies used were as follows: anti-GluR2 (Millipore), anti-LC3 (M152–3; MLB), A0024 (Daco), T22 (Millipore), Tau5 (Millipore), anti-pS396-tau (Invitrogen), anti-NDP52 (GeneTex), Alexa Fluor 488– and Alexa Fluor 568–conjugated secondary antibodies (Invitrogen), gold-conjugated (5 nm) secondary antibodies (British BioCell International), and horseradish peroxidase–conjugated secondary antibodies (Jackson ImmunoResearch Laboratories).

### In vivo electrical stimulation and fEPSP recording

The methods for the electrical stimulation and fEPSP measurement in in vivo preparations were based on ones described previously [20]. Briefly, each mouse was anesthetized with a 1.5% isoflurane–air mixture and fixed in a stereotaxic device (model 900, David Kopf Instruments). The skull of each mouse was exposed, and two holes were bored through the skull to reach the brain surface (with a center position of −1.75 mm from the bregma and 1.75 mm from the midline and −0.5 mm from the bregma and −0.5 mm from the midline). After a 1-h recovery period during which anesthesia was maintained, an electrode assembly and a cannula (#38 syringe tip) were inserted toward the stratum radiatum (projection area of Shaffer’s collaterals) of the hippocampus CA1 region and brain ventricle. The location of the electrode assembly was estimated based on the change in the form of the field excitatory postsynaptic potential (fEPSP) triggered by an electrical pulse with a duration of 100 µs.

When LFS-induced LTD was measured in vivo, 0.0333 Hz electrical stimulation (pulse duration = 100 s) was continuously applied from 1 h after the electrode position was fixed to monitor evoked fEPSPs and their stability. After stable fEPSPs (i.e., the ratio of the minimum to maximum slope was <0.8) were confirmed over a 15-min or longer period (fEPSPs were required to be stable for 1–5 h during the experimental condition), the baseline slope of the test stimulation–induced fEPSP was measured for 15 min. Then, after application of LFS (stimulation with 900 pulses at 1 Hz), the temporal change in the test stimulation–induced fEPSP was measured for 1 h. For this measurement, the electrical signal was amplified 100 times (ER-1; Cygnus Technology), digitized (Digidata 1321A; Axon Instruments), and processed on a computer. The amplitude and slope of each recorded fEPSP and fiber volley were measured by a custom application based on MATLAB (version 2013a, Mathworks Inc.). fEPSPs were analyzed only when the maximal amplitude was >1 mV, and the latency of the minimum peak from the stimulus was <7 ms.

When LFS-induced tau oligomerization was assessed in vivo, the schedule for the electrode and cannula penetration (1 h after the operative treatment), injection of chemical or vehicle solution (3.5 or 4.25 h after operative treatment), and LFS application (4 h after operative treatment) was fixed to normalize effects from anesthetization or operation. For the purpose of the experiment, LFS (900 pulses at 1 Hz) was applied twice successively (1800-pulse protocol) to enhance its influence. For intracerebral ventricle injection of solutions, a syringe pump (KTS310, Morumachi) was used. The injection speed was 1 μl/min, with a total injection volume of 3 μl. The solutions used consisted of 10 μM Bafilomycin in artificial cerebrospinal fluid (aCSF: 124 mM NaCl, 3 mM KCl, 26 mM NaHCO_3_, 1.25 mM NaH_2_PO_4_, 2 mM CaCl_2_, 1 mM MgSO_4_, and 10 mM d-glucose, bubbled with 95% O_2_/5% CO_2_) containing 1% DMSO or 10 mM 3MA in aCSF. Application of these solutions did not change the basic fEPSP amplitude (data not shown).

The electrode assembly used in this study consisted of two pairs of bipolar electrodes (stimulating and recording electrodes), which were made from tungsten or insulated nichrome wire. The tungsten wire, which had a polished end, was used to minimize physical damage to the tissue in which it was inserted. The nichrome wire, which had a gold-coated tip, was used to minimize the influence of metal elution on physiological responses. There were no notable differences in the stimulating effects on tau aggregation between the two wire types (data not shown).

### Acute slice preparations

For in vitro electrophysiology experiments, acute hippocampal slices were obtained from aged and adult mice. After decapitation, the brain was rapidly removed and placed in ice-cold aCSF with 1 mM kynurenic acid. Transverse hippocampal slices (350 **μ**m thick) were prepared using a Vibratome (VT1200S, Leica Biosystems). Hippocampal slices were stored in aCSF (20–25 °C) for 1–2 h before being transferred to the recording chamber, in which they were submerged in aCSF containing 20 μM picrotoxin at 32 °C with a flow rate of 2 ml/min. Picrotoxin, a GABA receptor antagonist, was used to reduce the effect of GABA-related effects. Extracellular field potentials were recorded in the CA1 region using glass electrodes containing aCSF. A stimulating electrode in CA2 was used to evoke fEPSPs with a test stimulus of a single pulse (15–20 μA constant current pulse inducing fEPSPs with a 50% amplitude relative to the maximum, 100-s duration, repeated at 30-s intervals). For this measurement, the electrical signal was amplified 100-fold (ER-1; Cygnus Technology), digitized (Digidata 1321A; Axon Instruments), and processed on a computer. The slope of the evoked fEPSP was measured using custom software based on MATLAB (version 2013a). In this experiment, one or two of the slices obtained from an individual mouse were used for drug application, and one or two of the remaining slices were used for vehicle application. Drug solutions and their respective vehicles were as follows: 0.1 μM Bafilomycin and aCSF containing 0.01% DMSO and 20 μM picrotoxin, 1 mM 3MA and aCSF containing 1% water and 20 μM picrotoxin, and 0.1 μM MG132 and aCSF containing 0.01% DMSO and 20 μM picrotoxin.

### Sarkosyl-insoluble (SI) fraction

Each isolated hippocampus was weighed and homogenized with 30 vol of cold HEPES-sucrose buffer (HSB: 320 mM sucrose; 4 mM HEPES; 2 mM EDTA, pH 7.4) with protease inhibitors (Sigma, diluted 1:100) and phosphatase inhibitors (Nacalai Tesque, diluted 1:100) and was centrifuged at 1000 g for 15 min at 4 °C to remove nuclear material and cell debris, resulting in the S1 fraction. Then, a high-salt sarkosyl fraction was obtained by adding an equal volume of extra-high-salt HSB (HSB with 1.6 M NaCl) with 2% sarkosyl solution to the S1 fraction. This mixture was incubated at 37 °C for 2 h and then was separated into the SI fraction and the sarkosyl-soluble (SS) fraction by centrifugation (200,000 g, 4 °C, 1 h).

### SDS-PAGE and western blotting

The methods of SDS-PAGE and western blotting were previously described [19]. In brief, each obtained fraction was analyzed by SDS-PAGE and western blotting. For SDS-PAGE, each fraction obtained was suspended in Laemmli sample buffer and subjected to SDS-PAGE using a 5–20% gradient gel (Wako). Separated proteins were blotted onto polyvinylidene difluoride (PVDF) membranes (GE Healthcare). The membranes were incubated with primary antibody (room temperature, 2 h) in Tris-buffered saline (TBS; 50 mM TrisHCl, 500 mM NaCl, pH 7.6), followed by the appropriate species of horseradish peroxidase–conjugated secondary antibody (room temperature, 30 min) in TBS. Chemiluminescent detection (ECL, GE Healthcare) was used for visualization. Quantification and visual analysis of immunoreactivity were performed with a computer-linked LAS-4000 Bio-Imaging Analyzer System (GE Healthcare). Antibody dilutions were as follows: A0024, 1:20,000; anti-ps395 tau, 1:1000; tau5, 1:500; anti-LC3, 1:1000; anti-NDP52, 1:1000; anti-GluA2, 1:1000; all secondary antibodies, 1:10,000.

### Blue native (BN)-PAGE and western blotting

For BN-PAGE analysis, each S1 fraction obtained from a hippocampus was subjected to centrifugation (12,500 g, 4 °C, 20 min) and divided into the crude synaptosomal (P2) fraction (i.e., the pellet), in which PSD-95 was largely recovered, and the synaptosome-depleted fraction (S2). The P2 fraction pellet was suspended in 50 μl native lysis buffer (NativePAGE Sample Prep Kit, Invitrogen) with 0.1% Triton X-100 and run on a Tris-Bis gradient gel (3–12% Bis-Tris Protein Gels, Novex). After blotting to a PVDF membrane using a transfer tank (Mini Blot Module, Novex), the tau oligomers labeled with tau oligomer–specific antibody T22 (diluted 1:500 in TBS) or anti-tau A0024 (diluted 1:20,000 in TBS) were visualized by a chemiluminescence method using the LAS-4000 system.

### Immunoprecipitation

To analyze the components of the molecular complex detected by T22, a commercial immunoprecipitation kit (direct magnetic IP/Co-IP kit, Pierce) was used. To bind T22 antibody on magnetic beads (NHS-activated magnetic beads**;** Pierce), 10 μg of magnetic beads was washed with ice-cold 1 mM HCl, and then 500 μl of T22 solution (10 μl T22 diluted in 500 μl TBS) was added, and the mixture was incubated at room temperature for 1 h. The beads were isolated with a magnet and washed with Elution buffer (Pierce), and the reaction was quenched by the addition of 3 M ethanolamine. Then each P2 fraction pellet, which had been diluted in 200 μl HSB with 0.5% Triton X-100, protease inhibitors (diluted 1:200), and phosphatase inhibitors (diluted 1:200), was exposed to the antibody-bound beads overnight at 4 °C. The beads were again isolated with a magnet and washed with HSB and then distilled water before being rinsed in 10 μl Elution buffer to recover tau oligomers. After neutralization by the addition of 1 μl of 2 M Tris solution, the resulting immunoprecipitated proteins were analyzed by SDS-PAGE and western blotting as described above.

### Electron microscopy

The morphological features of tau oligomers in the SI fraction were investigated by an immunogold electron microscopy technique [51]. In brief, the SI pellet obtained from each hippocampus was washed three times with TBS and resuspended in 50 μl TBS. For electron microscopy, the TBS solution was incubated with primary antibody (A0024, diluted 1:200 or T22, diluted 1:50) in TBS for 2 h at 4 °C. After washing, the samples were absorbed onto glow-discharged supporting membranes on 400-mesh grids and incubated with a 5-nm colloidal gold–conjugated secondary antibody (diluted 1:200) for 90 min. After fixing with 2% glutaraldehyde, grids were negatively stained with 2% sodium phosphotungstic acid, dried, and then examined with a transmission electron microscope (Tecnai 12, FEI). In examination using T22 antibody, SI samples suspended in TBS were directly exposed to a supporting membrane on a mesh and were immunostained with the same primary and secondary antibodies on the mesh after blocking with 0.05% bovine serum albumin (BSA) and 1% normal horse serum.

### Immunohistochemistry and immunofluorescence staining

Mice were deeply anesthetized with pentobarbital (50 mg/kg) and then transcardially perfused with 10% formalin. Brains were postfixed in the same fixative for 16 h and embedded in paraffin and sectioned (4–6 mm) in the coronal plane. Deparaffinized sections were treated with Target Retrieval Solution (Dako) for 20 min at 80 °C, blocked in 0.1% BSA/TBS, and incubated with primary antibodies (anti-LC3, 1:500; anti-GluA2, 1:1000) in 0.1% BSA/TBS overnight at 4 °C. Immunohistochemical staining was performed with immPRESS Reagent kit (Vector) and immPACT DAB (Vector). For fluorescent staining, slices were incubated with Alexa Fluor 488– and Alexa Fluor 568–conjugated secondary antibodies (1:500) in 0.1% BSA/TBS overnight at 4 °C. A LSM700 laser confocal microscope (Zeiss) was used for fluorescent observations.

### S**tatistical analysis**

In the present study, when the normalized levels of the SI tau in ipsilaterally stimulated hippocampi were compared to their internal controls (i.e., contralateral ones obtained from the same animals), the one-sample t-test against a theoretical value of ‘1’ was used because the normalized level of contralateral ones was logically ‘1’. When the normalized levels of the SI tau in stimulated hippocampi were compared to the ones in another group, unpaired *t*-test was mainly used. In other cases, we used unpaired *t*-test, paired *t*-test or two-ways ANOVA. These analyses were performed with Prism 7 (GraphPad Software, Inc.).

## Results

### Stimulus-inducible and age-dependent oligomerization of tau in the mouse brain

**Fig. 1:**
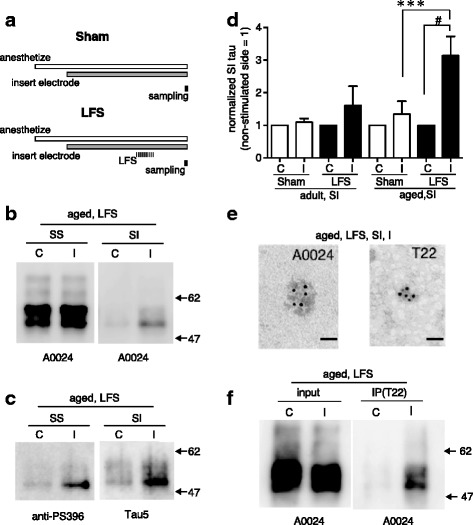
LFS-induced and age-dependent oligomerization of tau in wild-type mouse hippocampus. **a** Experimental schedules used in this study. In the LFS group, 1800 electrical pulses at 1 Hz were applied to one side of the hippocampus (Schaffer’s collateral area in the CA1) in anesthetized mice before hippocampus sampling. In the sham-control group, each mouse received the same operation (anesthetization, electrode penetration, test stimulation for determination of the insertion area) as the LFS group but did not receive LFS. **b**, **c** Typical western blot analysis of sarkosyl-soluble (SS) and sarkosyl-insoluble (SI) fractions obtained from a contralateral (C) and ipsilateral (I) hippocampus from an aged LFS mouse. Blots were analyzed for total tau expression with antibody A0024 (**b**) and Tau5 (**c**) and for phosphorylated tau with anti-PS396-tau **c**. **d** Graph showing the mean normalized tau levels (detected by using A0024) in SI fractions at ipsilaterally stimulated (I) and control (unstimulated) contralateral (C) hippocampi in the sham and LFS groups of adult and aged animals (adult sham, *n* = 4; adult LFS, *n* = 5; aged sham, *n* = 5; aged LFS, *n* = 8). ** *p* < 0.01, unpaired *t*-test; #*p* < 0.05, one-sample *t*-test against a theoretical value of ‘1’. **e** Typical electron microscopy images showing the morphology of tau aggregates from the SI fraction from hippocampi of aged LFS mice. Each black dot is an immunogold particle attached to the indicated tau antibodies. Bar: 20 nm. **f** LFS induced increases in oligomeric tau, which was immunoprecipitated (IP) by the T22 antibody from the ipsilateral side (I), but not the contralateral side (C), although such side-specific increases in precipitated tau were not detected in the total tau level of P2 fractions (input). A0024 was used for western blot analysis. These tendencies were confirmed in three independent experiments

We applied low-frequency stimulation (LFS; 1800-pulse stimulation at 1 Hz) to one side of the hippocampus (i.e., the ipsilateral side) of wild-type mice to induce LTD and then collected both the ipsilateral and contralateral sides of the hippocampus 30 min after stimulation was stopped for biochemical analysis (Fig. 1a). In the aged mice (20–24 months old), SI tau was detected mainly in the ipsilateral hippocampus (Fig. 1b), which had received LFS directly, but not in the contralateral hippocampus (Fig. 1b, c), although there was no strong difference between the ipsilateral and contralateral hippocampus in the sarkosyl-soluble fraction (Fig. 1b, SS). Western blot analysis also showed that SI tau was phosphorylated at Ser396 (Fig. 1c), which is a critical phosphorylation site relating to LTD [37].

To evaluate the stimulating effect, we measured the SI tau level of the ipsilateral and the contralateral hippocampus in each animal and calculated the normalized tau levels for both sides by dividing by the contralateral level. Note that the normalized tau level in the contralateral side is thus always ‘1’. In the animals receiving the sham operation, in which all steps except LFS were carried out (Fig. 1a, Sham), the normalized level of SI tau in ipsilateral hippocampi was 1.098 ± 0.1099 a.u. (mean ± SEM; *n* = 4) in adult mice (Fig. 1d, adult Sham I) and 1.342 ± 0.4007 (*n* = 5) in aged ones (Fig. 1c, aged Sham I; see also Additional file 1: Figure S1). The statistical analysis showed no significant difference (*p* = 0.4420, one-sample *t*-test against a theoretical value of ‘1’) between these ipsilateral (stimulated) hippocampi and their contralateral (unstimulated) controls, indicating that the operation steps other than LFS did not have a significant effect on SI tau. In contrast, the quantitative analysis showed that in the aged hippocampi there was significant enhancement of SI tau (*p* = 0.0123, one-sample *t*-test) in the LFS-stimulated ipsilateral sides (normalized level = 3.143 ± 0.5846, *n* = 8) relative to the contralateral ones (Fig. 1d, aged LFS C and I). Therefore, we concluded that LFS has an important role in the formation of SI tau. This is supported by the statistically significant difference (*p* = 0.0486, unpaired t-test) in the normalized SI tau levels of the aged ipsilateral hippocampus groups corresponding to sham-operated and LFS-stimulated mice (Fig. 1d, aged Sham I and LFS I). Furthermore, in adult mice, there was no LFS-induced increase in SI tau in the ipsilateral hippocampi (Fig. 1d, adult LFS I) relative to contralateral hippocampi (*p* = 0.3621, one-sample *t*-test) or to sham-operated ones (*p* = 0.4863, unpaired *t*-test). Thus, the increase in SI tau in stimulated hippocampi is age dependent.

Next, the morphology of tau aggregates in the SI fraction, which were visualized by immunogold labeling, was examined by electron microscopy. In the SI pellet obtained from aged LFS mice, although there were very few fibrillar aggregates, such as paired helical fibers or straight filaments, we found many granular oligomers that were recognized by the pan-tau antibody A0024 or the oligomeric-tau antibody T22 (Fig. 1e). Thus, these data suggest that SI tau originates from granular oligomers.

To confirm the LFS-induced oligomerization of tau, an analysis using oligomer-selective tau antibody (T22) was performed. Blue native electrophoresis showed that the T22 signal in crude synaptosomal fractions (P2) from aged LFS hippocampi was mainly observed above 500 kDa and that LFS shifted the signal upwards as compared with the signal observed for the unstimulated (contralateral) side (Additional file 1: Figure S2). These findings strongly suggest that LFS induced oligomerization of tau. An immunoprecipitation experiment also demonstrated that LFS increased tau oligomers captured by T22 from the ipsilateral side, but not from the contralateral side, although there was little difference in total tau levels between both sides (Fig. 1f). These results indicate that LFS formed tau oligomers in stimulated neurons in the aged hippocampus.

### Age-dependent alteration in a mechanism supporting LTD

**Fig. 2:**
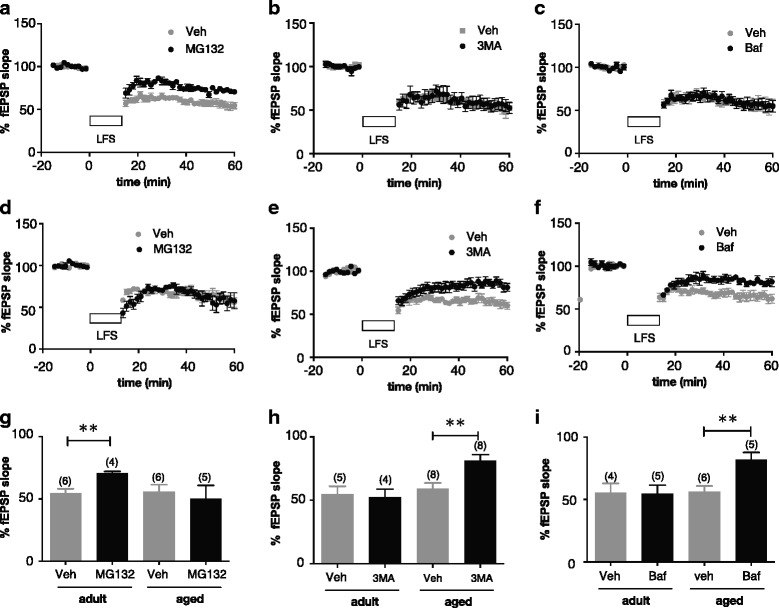
Age-dependent alteration of protein degradation mechanisms that support LTD formation. **a**–**f** The effects of several protein degradation blockers on LTD were examined in hippocampus slices from 4- to 8-month old adult **a**–**c** and 20- to 24-month old aged **d–f** mice. After being sliced, each slice was placed in a chamber filled with aCSF containing 20 μM picrotoxin and incubated for 3 h at 33 °C. Slices were then exposed to the following blockers or vehicle control (veh) in aCSF with picrotoxin for 30 min before the start of LFS (900 pulses, 1 Hz): 0.1 μM MG132 **a**, **d**, 1 mM 3MA **b**, **e**, and 0.1 μM Bafilomycin **c**, **f**. In this experiment, we used a 900-pulse protocol for LTD induction, which led to the formation of stable LTD in slice preparations (data not shown), to avoid any reduction in sensitivity to chemicals that would result from saturated LTD. In control experiments, these concentrations did not directly change the field excitatory postsynaptic potential (fEPSP) size (data not shown). Each graph shows temporal change in normalized fEPSP slop before and after LFS application. Data are shown as the mean ± SEM. **g**–**i** The effect of LFS on fEPSP was statistically analyzed based on the average data obtained from 59 to 60 min in the absence and presence of MG132 **g**, 3MA **h**, and Bafilomycin **i**. **p < 0.01, unpaired t-test. Data are shown as the mean ± SEM. The number of slices used in each group is shown in parentheses above each column

Age-dependent changes in the mechanism of LFS-induced LTD were examined to reveal the molecular substrates controlling LFS-induced oligomerization of tau in aged brains. In both in vivo and in vitro slice preparations, we found no significant difference between adult (5–10 months old) and aged (20–24 months old) mice with respect to LFS-induced LTD (see Fig. 2g–i adult and aged vehicle, and Additional file 1: Figure S3c adult and aged *Mapt* ^+/+^). Although age dependency of the tau-deficit effect on LTD was also examined, there was no significant change with aging (Additional file 1: Figure S3a-c *Mapt* ^+/−^ *Mapt* ^−/−^).

More detailed analyses were performed using slice preparations obtained from mice of different ages (4- to 8-month-old adults and 20- to 24-month-old aged mice), as age-dependent changes in proteasome activity in the hippocampus have been reported [17, 40]. We focused on the protein degradation mechanism that supports LTD [7, 13]. LFS-induced LTD in slice preparations from adult mice was partially inhibited by MG132 (Fig. 2a, g adult), whereas the MG132 did not affect LTD in slice preparations from aged mice (Fig. 2d, g aged). These results indicate an age-dependent decrease in the contribution of MG132-sensitive cascades, such as proteasome cascades [32, 47], to LTD induction. In parallel with this change, we found that two ALP inhibitors, 3-methyladenine (3MA) [34], which prevents the formation of autophagosomes [54], and Bafilomycin [21, 36], which attenuates fusion of lysosomes to autophagosomes [29], reduced the LTD level in slice preparations from aged, but not adult mice (Fig. 2b–i), suggesting an age-dependent increase in the contribution of the ALP to LTD. Thus, our findings strongly suggest that protein degradation systems supporting the LTD switch from MG132-sensitive systems (e.g., the proteasome pathway) to 3MA- and Bafilomycin-sensitive systems (e.g., ALP) in response to aging.

### LFS-induced tau oligomerization is associated with autophagy activity

**Fig. 3:**
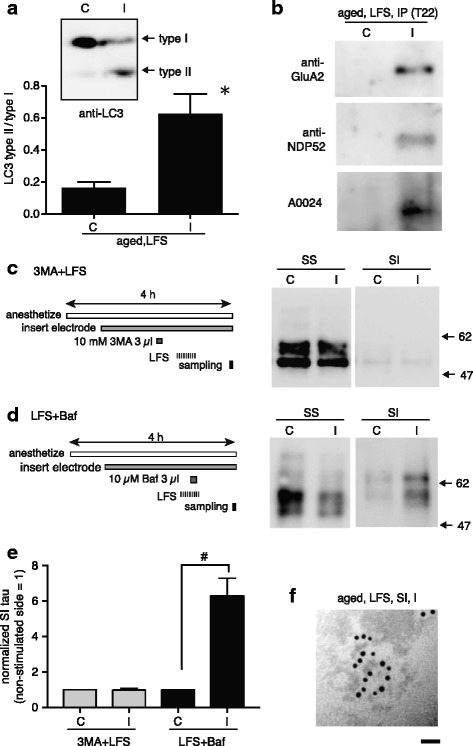
The ALP in aged neurons contributes not only to LTD but also to the formation of tau oligomers. **a** LFS (1800 pulses, 1 Hz) reduced type I LC3 and increased type II LC3 mainly in the ipsilateral side of the aged hippocampus (immunoblot inset), resulting in a significant difference in the type II/type I LC3 ratio between the stimulated (ipsilateral, I) and contralateral (control) side (C) of the hippocampus (graph; *n* = 4, *p* = 0.0116, paired *t*-test). **b** Immunoprecipitation of LFS-induced tau oligomers by T22 indicated the presence of GluA2 (*top blot*) and NDP52 (*middle blot*) in addition to tau (*bottom blot*) in the molecular complexes obtained from the stimulated side (I) after LFS. Within the control (contralateral, C) side, the molecular complex was barely detected. These tendencies were confirmed in three independent experiments. **c** Intracerebral ventricle application of 3MA (1 μl/min of a 10 mM solution for 3 min) was carried out from 15 min before LFS (1800 pulses, 1 Hz), preventing LFS-induced tau oligomerization. The left panel shows the experimental procedure. The *right* panel shows an example of western blot analysis of sarkosyl-soluble (SS) and sarkosyl-insoluble (SI) fractions using A0024 tau antibody. **d** When Bafilomycin was applied (1 μl/min of a 10 μM solution for 3 min) from 15 min after the start of LFS (after 900 of 1800 pulses), LFS-induced tau oligomerization was not prevented, but 60- to 64-kDa hyperphosphorylated tau was formed. **e** Summary of normalized-tau levels in SI fractions from ipsilateral and contralateral hippocampi, which were obtained after LFS plus 3MA or Bafilomycin application (*n* = 5 for each group). Data are shown as the mean ± SEM. #*p* < 0.05 for a one-sample *t*-test against a theoretical value of ‘1’. **f** An electron microscopy image showing the morphology of immunogold-labeled tau oligomers formed by LFS under Bafilomycin treatment. Bar: 10 nm. In all experiments described here, aged wild-type mice (20–24 months old) were used

On the basis of the data presented above, it is likely that aging altered a component of the LTD mechanism, which led to an increase in LFS-induced tau oligomerization. To elucidate the influence of an enhanced ALP on LFS-induced tau oligomerization in aged hippocampus, we examined, as a first step, whether LFS promoted the ALP in aged brains. The ratio of type II LC3 to type I LC3, which is an indicator of the formation of autophagosomes, was assessed by quantification of western blots. The LFS (ipsilateral) hippocampus showed a higher type II/type I ratio (Fig. 3a, I) than the contralateral one (Fig. 3a C) with a statically significant difference (*p* = 0.0116, paired *t*-test). Such enhancement of the type II/type I ratio by LFS was not detected in the sham-operated hippocampi (Additional file 1: Figure S4). Therefore, LFS-induced potentiation of autophagy was confirmed in the aged mouse brain.

As a second step, we investigated the association between LFS-induced autophagy and LFS-induced tau aggregation by using co-immunoprecipitation. The molecular assembly immunoprecipitated by T22, which contained NDP52 and GluA2 in addition to tau, was found only in hippocampi that received LFS (Fig. 3b, I) but not in the contralateral control (Fig. 3b, C). NDP52 is a bridging protein that connects proteins to LC3-localizing autophagosomes and contributes to capturing phosphorylated tau on the autophagosome [46]. These results suggest that the formation of tau oligomers occurs through the degradation or recycling process of GluA2-containing AMPARs via the ALP in aged hippocampal neurons.

Third, we examined the influence of an early-phase inhibitor of the ALP, 3MA, upon LFS-induced tau oligomerization. In this experiment, the inhibitor was applied to the anterior part of the lateral ventricle 30–60 min before LFS in aged mice to perturb the early phase of the ALP, and the hippocampi were collected 30 min after the end of LFS (Fig. 3c, left illustration). In these 3MA-treated brains, SI tau, which was strong enhanced in the ipsilateral hippocampus in the absence of 3MA (see Fig. 1b, SI I), was barely noticeable (Fig. 3c, SI I). That is, the normalized level of SI tau in the ipsilateral hippocampi was close to one, and there was no significant difference between the ipsilateral and contralateral side (*p* = 0.8960, one-sample *t*-test) (Fig. 3e, 3MA + LFS, I and C). Thus, this suggests that LFS-induced formation of autophagosomes is critical for LFS-induced oligomerization of tau in vivo. In addition, immunofluorescence analysis showed the localization of phosphorylated tau on LC3 puncta within dendrites (Additional file 1: Figure S5), suggesting the accumulation of tau on autophagosomes in the aged hippocampal neurons.

Finally, to analyze the prevention effect of the late phase of the ALP on the LFS-induced oligomerization of tau, Bafilomycin was applied after a 30-min delay from the start of LFS (Fig. 3d, left illustration). Bafilomycin did not cancel the effect of LFS on tau aggregation in the ipsilateral hippocampus (normalized level of SI tau = 6.302 ± 2.226, n = 5, *p* < 0.05, one-sample *t*-test) (Fig. 3d and e), but it did increase the level of SI tau (*p* < 0.05, unpaired *t*-test) as compared with untreated ipsilateral hippocampi that received LFS (Fig. 1d, aged LFS I). Interestingly, western blotting indicated that the SI tau aggregates mainly consisted of 60- and 64-kDa tau (Fig. 3d), which are known as highly phosphorylated or hyper-phosphorylated tau [16] and are rarely observed after LFS application under normal conditions (see Fig. 1b). Therefore, these findings indicate that the late phase of autophagy is involved not only in the degradation of tau oligomers but also in preventing the formation of hyper-phosphorylated tau aggregates.

Formation of the hyper-phosphorylated 64-kDa tau involves the generation of fibrillar tau aggregates, such as paired helical fibers or NFTs, in mouse brains that over-express human tau [41, 42]. To elucidate the influence of hyper-phosphorylation on the morphology of SI tau aggregates, we analyzed the SI fraction obtained after LFS under Bafilomycin by electron microscopy. Hippocampi exposed to Bafilomycin during LFS frequently showed the fibrillar form of tau aggregates (Fig. 3f) in addition to the granular form, which was observed in SI fractions obtained after LFS without Bafilomycin (Fig. 1e). Thus, these findings suggest that the protein-degradation ability of the ALP strongly influences the phosphorylation state of LFS-induced tau oligomers and their morphology.

## Discussion

The present study showed that LFS-induced LTD in aged hippocampus is critically dependent on the throughput level of the 3MA- and Bafilomycin-sensitive pathway but not on the MG132-sensitive pathway. This suggests that a part of the LTD cascade switches with age from a proteasome-sensitive to autophagy-sensitive one. Contributions of the MG132-sensitive pathway on NMDA-induced, AMPA-induced, and electrical stimulus–induced internalization of AMPARs have been described (see [13]). In contrast, NMDA application triggers autophagy, which contributes to AMPAR internalization and/or trafficking in cultured hippocampal neurons, although the physiological role of NMDA-induced autophagy is unclear [46]. These findings suggest that neurons are able to use those different protein-degradation pathways to control AMPAR internalization. Age-dependent reduction of proteasome activity in the hippocampus, cortex, and spinal cord in rats has been described [18]. Thus, it is possible that the alteration influences neuronal selection of a protein-degradation pathway relating to LFS-induced LTD. At a minimum, the age-dependent alteration in the response to the drugs used in this study represents an age-dependent change in the dynamic properties of the protein degradation system, which is required for LTD.

Meanwhile, the present study demonstrates the importance of the switching of the protein-degradation pathway in an age-dependent manner relative to the risk of tauopathy formation, because, as shown here, LFS induces not only LTD but also tau aggregation in the aged hippocampus. Similar to LTD formation in the aged mice, tau aggregation was also sensitive to the ALP, especially during the process of autophagosome formation. It was reported that the autophagic marker p62/SQSTM1 accumulates and colocalizes with hyperphosphorylated tau in human tauopathies [35]. In addition, studies that analyzed the aggregation mechanism of pro-aggregate mutant tau have also suggested the importance of chaperone-mediated autophagy for the formation of tau aggregates [8, 53]. As there are notable differences between those studies and our study with respect to the tau species used and the experimental system, the results cannot be directly compared. However, those independent studies lead to a similar conclusion that the early stage of tau aggregation in vivo requires a certain protein accumulation mechanism.

Extracellular tau detected in human cerebrospinal fluid increases in an age-dependent and pathogenesis-dependent manner [15, 48]. Analysis of interstitial fluid in a tauopathy mouse model has shown an extracellular secretion of tau aggregates [50]. Moreover, extracellular tau oligomers are taken up by neurons via endocytosis mechanisms and contribute to tau pathology [10]. If tau oligomers form within autophagosomes or lysosomes as a result of LFS, they could be candidates for subsequently becoming extracellular tau aggregates, because tau aggregates within intracellular vesicles are secreted extracellularly and can propagate to other cells [15]. In contrast, as in the case of tau aggregation during chaperone-mediated autophagy [8, 53], there is the possibility that tau aggregation occurs on the lysosomal/phagosomal surface. In this case, the LTD-ALP cascade may be involved more directly in cytoplasmic pathogenesis during tauopathy. Therefore, the early phase of the LTD-ALP cascade potentially acts as a supplier of tau aggregates in aged brains, which contributes to the pathogenesis in direct and/or indirect ways.

The analysis of AD model mice has demonstrated that over-expressing mutant Aβ alters electrical activity patterns in the brain [1, 28] and shifts the characteristics of synaptic plasticity (i.e., long-term potentiation is down-regulated and LTD is up-regulated) [14, 38]. Thus, the toxic form of Aβ potentially enhances the tau-aggregation cascade that accompanies LTD. In AD model mice, neurons that accumulate Aβ oligomers show impaired proteasome activity [45] and enhanced activation of autophagy [6]. Therefore, it is possible that those AD-related factors may influence tau aggregation via the LTD-ALP cascade.

## Conclusions

In the present study, we examined the physiological conditions related to OR demonstrated one of the physiological conditions that result in the formation of (SI-insoluble) tau oligomers in wild-type mouse neurons. A part of the LFS-induced LTD cascade of hippocampal neurons is spontaneously altered during aging, resulting in increased contribution of autophagy to that process and enhanced tau oligomerization triggered by LFS. The pharmacological experiments suggest the requirement of autophagosome formation accompanied by LFS for tau oligomerization in aged neurons. The age-dependent and autophagosome-dependent tau oligomerization that occurs with synaptic depression might lead to the formation of pathological aggregates, which are accompanied by synaptic impairment, in tauopathies.

## Additional file

Additional file 1: Figure S1. Examples of western blots that analyzed sarkosyl-soluble (SS) and -insoluble (SI) fractions obtained from hippocampi of sham-operated or LFS-applied wild-type mice from the adult (8 months old) and aged (23 months old) groups. Tau was visualized by the pan-tau antibody A0024. In many of the sham-operated mice, SI tau was detected in neither the contralateral (C) nor ipsilateral (I) side of the hippocampi, although a weak signal was observed in both sides in a minority of cases. Aged LFS mice tended to show a stronger tau signal in the SI fractions from the stimulated side of the hippocampus. **Figure S2.** Tau oligomers in P2 crude synaptic fractions were analyzed by western blotting using the tau oligomer–selective antibody T22 after blue native electrophoresis. This representative blot shows oligomers in the ipsilateral side (I) and contralateral side (C) of LFS hippocampi from an aged (24 months old) mouse, demonstrating increased high-molecular-weight oligomers in the stimulated hippocampus. **Figure S3.** To look for age-dependent changes in LFS-induced LTD and the effect of tau knockout upon it, in vivo LTD was examined in six groups of mice: adults (5–10 months old) and aged (20–24 months old) groups of wild-type (*Mapt* ^+/+^), tau-knockout heterozygous (*Mapt* ^+/−^) and tau-knockout homozygous (*Mapt* ^−/−^) mice. (**a, b**) In both adult (**a**) and aged (**b**) groups, LFS (900 pulses, 1 Hz) effectively reduced the fEPSP amplitude (slope) for at least 60 min in wild-type mice, but not in tau-knockout heterozygous or homozygous mice. (**c**) Two-way ANOVA using data obtained at 60 min after starting LFS indicated a strong effect of tau knockout on LTD performance (F(2, 29) = 13.25, *p* < 0.0001), but no influence of age on this effect (F(1, 29) = 0.09874, *p* = 0.7556). Each number in parentheses in the graph shows the number of animals for each group. Data are shown in each graph as the mean ± SEM. **Figure S4.** Comparison of the ratio of LC3 type II, an active form of LC3, to LC3 type I in the hippocampi from sham-operated aged (20–24 months old) mice. (*a*) A representative western blot showing the state of LC3 in the hippocampus from a mouse that received the sham operation (see Fig. 1a, Sham) on the ipsilateral side (I), along with the contralateral control (C). (*b*) Quantitative comparison of the ratio of type II to type I LC3 showed no difference in the LC3 state between the ipsilaterally stimulated hippocampi and the control ones after the operation (*p* = 0.4966, paired *t*-test, *n* = 3). Data are shown as the mean ± SEM. **Figure S5.** Intracellular localization of phosphorylated tau (PS396) and its association with LC3 puncta. Hippocampi were fixed and sampled from a 24-month-old aged mouse after LFS and injection of Bafilomycin (see schematic for timeline in Fig. 3d) and were sliced parallel to the sagittal plane at a thickness of 5 μm after embedding with paraffin. Following deparaffinization, the distribution of phosphorylated tau (anti-pTau; PS396) was examined using immunohistochemical and immunofluorescence methods. (**a**) Immunohistochemical examination showed an increase in dot-like immunoreactivity in the somato-dendritic portion of CA1 pyramidal neurons in the stimulated (ipsilateral; I) side of the hippocampus as compared with the unstimulated (contralateral; C) one. (**b**) This dot-like distribution of phosphorylated tau was confirmed by immunofluorescence. Importantly, the laser confocal analysis showed co-localization of the anti-LC3 (green) and anti-pTau (red) signal (yellow, Merged). This suggests that phosphorylated tau accumulates at autophagosomes labeled by LC3. str. Pyr., stratum pyramidale in the CA1 region; str. Rad., stratum radiatum in the CA1 region (PDF 939 kb)
