# Supplementary figures and images for: Age-dependent changes in synaptic plasticity enhance tau oligomerization in the mouse hippocampus
Source: Acta Neuropathol Commun. 2017 Sep 6;5:67. doi: 10.1186/s40478-017-0469-x (PMC5586024; doi:10.1186/s40478-017-0469-x)

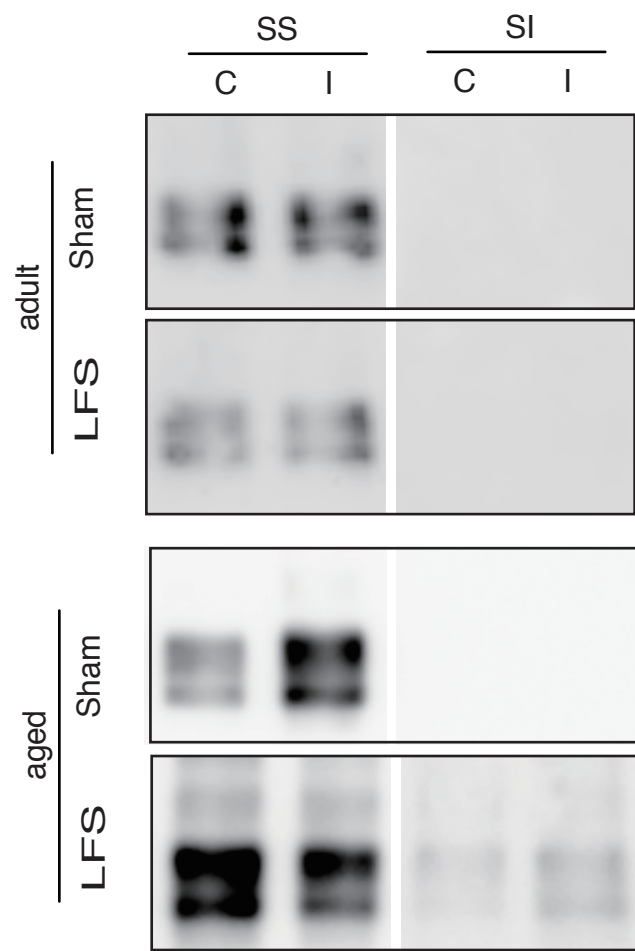

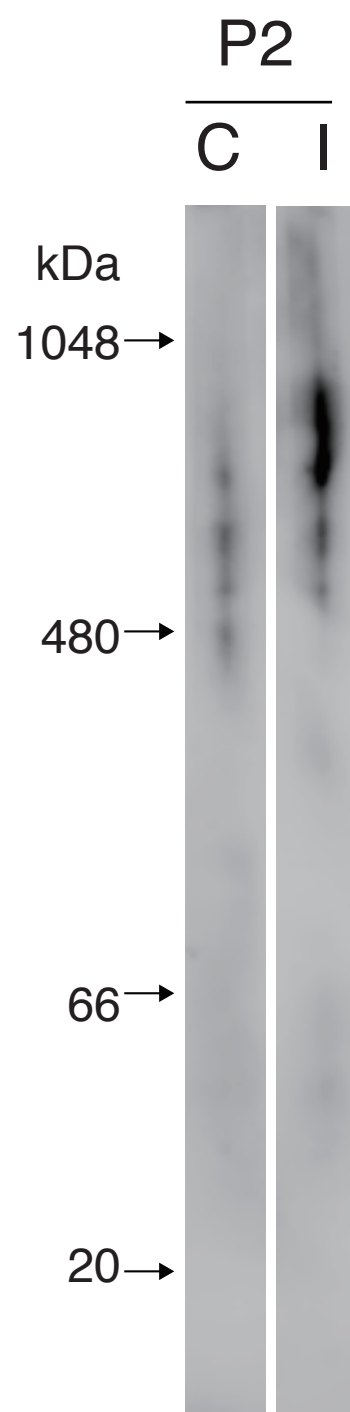

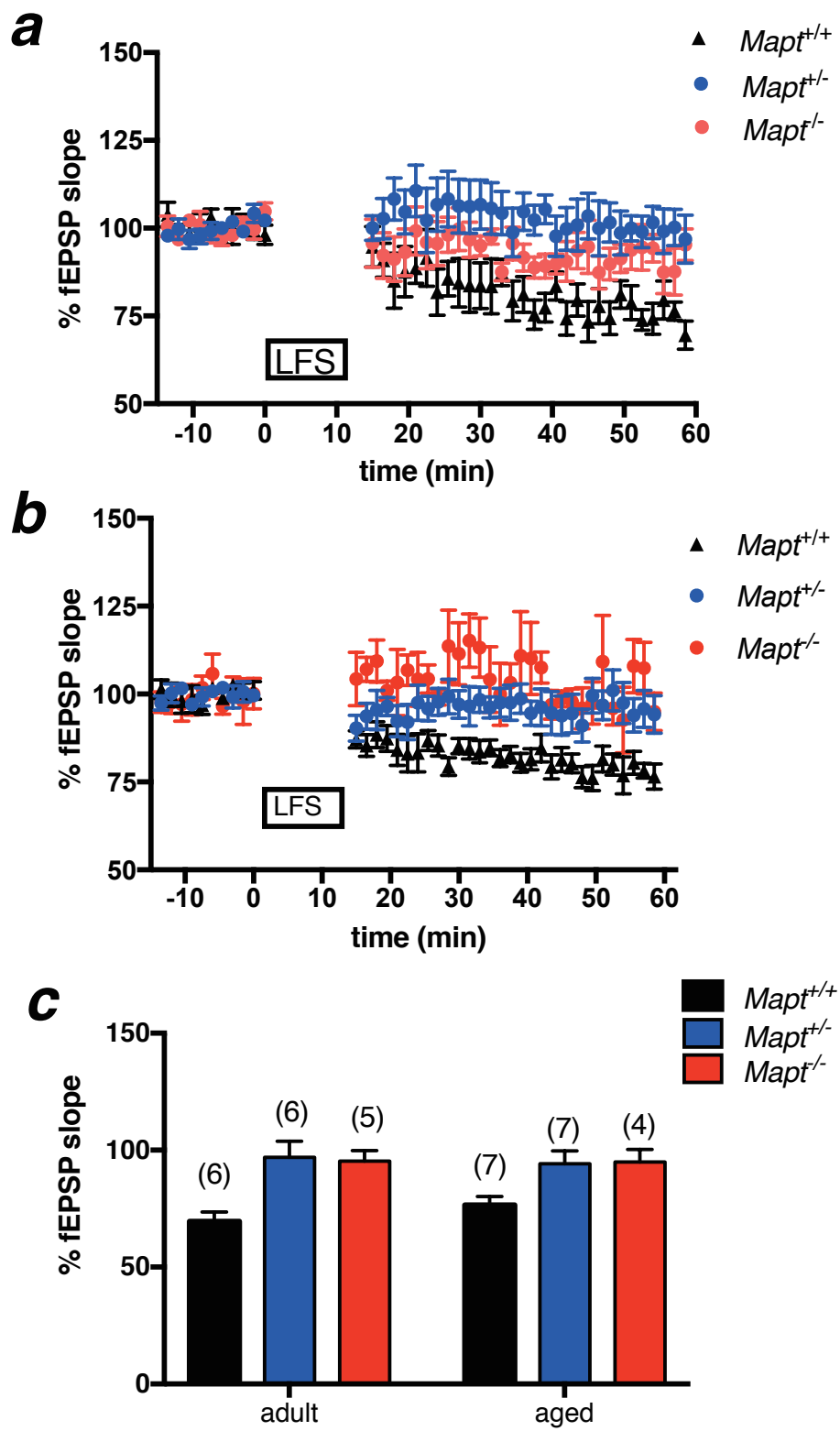

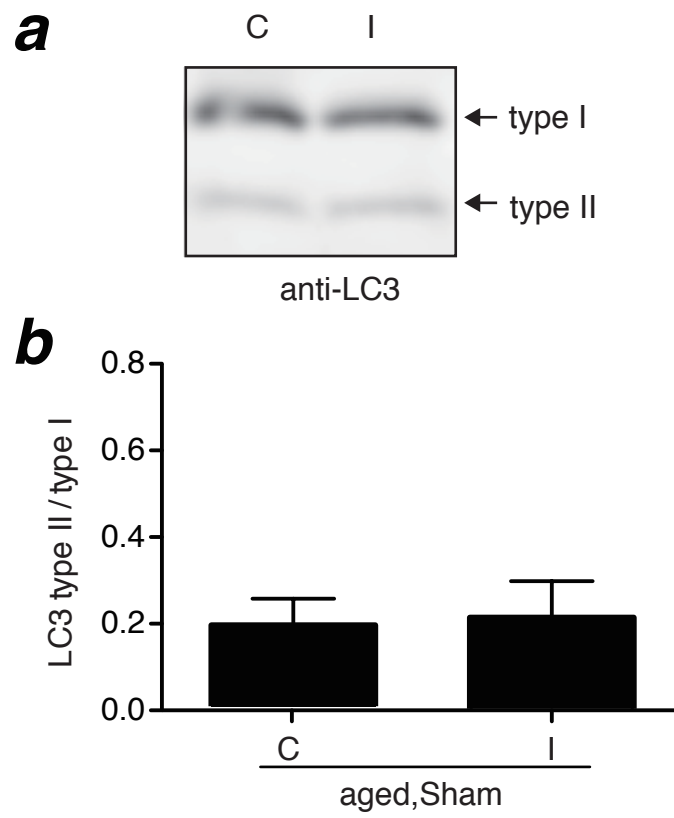

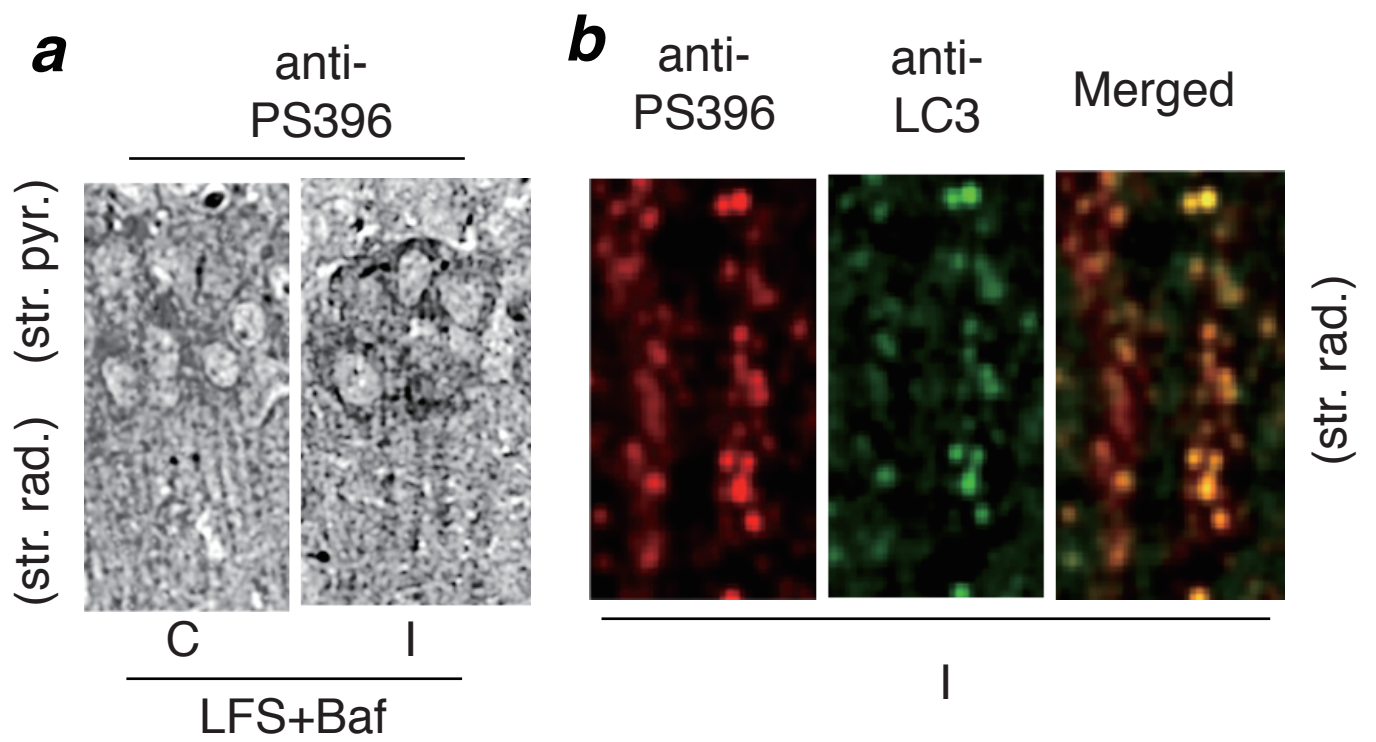

Supplement: Additional file 1: Figure S1. — Examples of western blots that analyzed sarkosyl-soluble (SS) and -insoluble (SI) fractions obtained from hippocampi of sham-operated or LFS-applied wild-type mice from the adult (8 months old) and aged (23 months old) groups. Tau was visualized by the pan-tau antibody A0024. In many of the sham-operated mice, SI tau was detected in neither the contralateral (C) nor ipsilateral (I) side of the hippocampi, although a weak signal was observed in both sides in a minority of cases. Aged LFS mice tended to show a stronger tau signal in the SI fractions from the stimulated side of the hippocampus. Figure S2. Tau oligomers in P2 crude synaptic fractions were analyzed by western blotting using the tau oligomer–selective antibody T22 after blue native electrophoresis. This representative blot shows oligomers in the ipsilateral side (I) and contralateral side (C) of LFS hippocampi from an aged (24 months old) mouse, demonstrating increased high-molecular-weight oligomers in the stimulated hippocampus. Figure S3. To look for age-dependent changes in LFS-induced LTD and the effect of tau knockout upon it, in vivo LTD was examined in six groups of mice: adults (5–10 months old) and aged (20–24 months old) groups of wild-type (Mapt +/+), tau-knockout heterozygous (Mapt +/−) and tau-knockout homozygous (Mapt −/−) mice. (a, b) In both adult (a) and aged (b) groups, LFS (900 pulses, 1 Hz) effectively reduced the fEPSP amplitude (slope) for at least 60 min in wild-type mice, but not in tau-knockout heterozygous or homozygous mice. (c) Two-way ANOVA using data obtained at 60 min after starting LFS indicated a strong effect of tau knockout on LTD performance (F(2, 29) = 13.25, p < 0.0001), but no influence of age on this effect (F(1, 29) = 0.09874, p = 0.7556). Each number in parentheses in the graph shows the number of animals for each group. Data are shown in each graph as the mean ± SEM. Figure S4. Comparison of the ratio of LC3 type II, an active form of LC3, to LC3 type I in the h [file 40478_2017_469_MOESM1_ESM.pdf]
